# Offer of primary care services and detection of tuberculosis incidence in Brazil

**DOI:** 10.11606/S1518-8787.2018052000131

**Published:** 2018-05-03

**Authors:** Daniele Maria Pelissari, Patricia Bartholomay, Marina Gasino Jacobs, Denise Arakaki-Sanchez, Davllyn Santos Oliveira dos Anjos, Mara Lucia dos Santos Costa, Pauline Cristine da Silva Cavalcanti, Fredi Alexander Diaz-Quijano

**Affiliations:** I Ministério da Saúde. Coordenação Geral do Programa Nacional de Controle de Tuberculose. Brasília, DF, Brasil; II Ministério da Saúde. Coordenação Geral de Acompanhamento e Avaliação da Atenção Básica. Brasília, DF, Brasil; III Fundação Oswaldo Cruz. Centro de Pesquisa Aggeu Magalhães. Programa de Pós-Graduação em Saúde Coletiva. Recife, PE, Brasil; IV Universidade de São Paulo. Faculdade de Saúde Pública. Departamento de Epidemiologia. São Paulo, SP, Brasil

**Keywords:** Tuberculosis, epidemiology, Tuberculosis, diagnosis, Primary Health Care, Health Care Quality, Access, and Evaluation, Tuberculose, epidemiologia, Tuberculose, diagnóstico, Atenção Primária à Saúde, Qualidade, Acesso e Avaliação da Assistência à Saúde

## Abstract

**OBJECTIVE:**

To evaluate the association between the health services offered by primary care teams and the detection of new tuberculosis cases in Brazil.

**METHODS:**

This was an ecological study covering all Brazilian municipalities that registered at least one new tuberculosis case (diagnosed between 2012 to 2014 and notified in the Information System of Notifiable Diseases) and with at least one primary care team evaluated by the second cycle of the National Program for Improving Access and Quality of Primary Care (PMAQ-AB). The variables of the PMAQ-AB were classified as proximal or distal, according to their relation with the tuberculosis diagnosis. Then, they were tested hierarchically in multiple models (adjusted by States) using negative binomial regression.

**RESULTS:**

An increase of 10% in the primary health care coverage was associated with a decrease of 2.24% in the tuberculosis detection rate (95%CI -3.35– -1.11). Regarding the proximal variables in relation to diagnosis, in the multiple model, the detection of tuberculosis was associated with the proportion of teams that conduct contact investigation (increase in Incidence Rate Ratio [IRR] = 2.97%, 95%CI 2.41–3.53), carry out tuberculosis active case finding (increase in IRR = 2.17%, 95%CI 1.48–2.87), and request culture for mycobacteria (increase in IRR = 1.87%, 95%CI 0.98–2.76).

**CONCLUSIONS:**

The variables related to the search actions were positively associated with the detection of new tuberculosis cases, which suggests a significant contribution to the strengthening of the sensitivity of the surveillance system. On the other hand, primary care coverage was inversely associated with the tuberculosis detection rate, which could represent the overall effect of the primary care on transmission control, probably from the identification and early treatment of cases.

## RESUMO

**OBJETIVO:**

Avaliar a associação entre os serviços de saúde ofertados por equipes de atenção básica e a detecção de casos novos de tuberculose no Brasil.

**MÉTODOS:**

Estudo ecológico, abrangendo todos os municípios brasileiros que registraram pelo menos um caso novo de tuberculose (diagnosticado entre 2012 a 2014 e notificado no Sistema de Informação de Agravos de Notificação) e com pelo menos uma equipe de atenção básica avaliada pelo segundo ciclo do Programa Nacional de Melhoria do Acesso e da Qualidade da Atenção Básica (PMAQ-AB). As variáveis do PMAQ-AB foram classificadas como próximais ou distais, segundo a sua relação com o diagnóstico de tuberculose. Em seguida, foram testadas hierarquicamente em modelos múltiplos (ajustados por Unidade Federada), usando regressão binomial negativa.

**RESULTADOS:**

Um incremento de 10% na cobertura da atenção básica esteve associado à redução de 2,24% na taxa de detecção de tuberculose (IC95% -3,35– -1,11). No que se refere às variáveis proximais ao diagnóstico, no modelo múltiplo, a detecção da tuberculose esteve associada à proporção de equipes que: realizam vigilância de contatos (incremento na Razão de Taxas de Incidência [RTI] = 2,97%; IC95% 2,41–3,53); fazem busca ativa de casos de tuberculose (incremento na RTI = 2,17%; IC95% 1,48–2,87); e, ofertam cultura para micobactérias (incremento na RTI = 1,87%; IC95% 0,98–2,76).

**CONCLUSÕES:**

As variáveis relacionadas às ações de detecção estiveram positivamente associadas à detecção de casos novos de tuberculose, sugerindo uma contribuição significativa ao fortalecimento da sensibilidade do sistema de vigilância. Por outro lado, a cobertura da atenção básica esteve inversamente associada à taxa de detecção de tuberculose, o que poderia representar o efeito global da atenção básica sobre o controle da transmissão, provavelmente, através da identificação e tratamento precoce de casos.

## INTRODUCTION

A total of 66,796 new tuberculosis (TB) cases were registered in Brazil in 2016, resulting in an incidence rate of 32.4/100,000 population^a^. Although the diagnosis of the disease is free of charge in the country, 13% of the people with the disease were not detected in Brazil that year according to the World Health Organization (WHO)^b^. The unidentified and, consequently, untreated cases are reservoirs of the disease and, therefore, a challenge for its control^1^.

The estimated population coverage of primary care (PC) was 69.3% in 2013^c^. In that same year, 61.4% of the new bacillary pulmonary tuberculosis cases were diagnosed and treated by PC services in Brazilian capitals^2^. However, in the Northeast region, 46.5% of the users sought the PC services in the early signs and symptoms of TB^3^. In Pelotas, state of Rio Grande do Sul, Brazil, 24.4% of the symptomatic respiratory patients sought PC, of which less than half had access to laboratory tests for TB diagnosis, from the lack of exam request by health professionals^4^.

Among the factors associated with the demand for these services in Brazil are the proximity of PC services, the waiting time for the appointment, and the relationship with the health team^3^ ^–^ ^5^. The provision of supplies and human resources^6^ and the presence of family health strategy (FHS) teams in health units^7^ are positively associated with case detection. In Ethiopia, delayed diagnosis was associated with non-knowledge about TB and the distance of the health service from the patient's residence^8^. Similarly, a meta-analysis of studies carried out in China has identified, among other factors, that living away from health services (rural area) was associated with delayed TB detection^9^.

In Brazil, the National Program for Improving Access and Quality of Primary Care (PMAQ-AB) aims to evaluate the improvement of care provided to the population by the PC. This evaluation covers specific aspects of TB, in particular TB detection^d^. National studies regarding the factors associated with the TB detection cases can generate evidence for the strengthening of public health policies. Thus, PMAQ-AB becomes a robust data source for this type of study, both because of its national coverage and because it is a qualified tool to evaluate several dimensions of the care provided for persons with TB.

In this context, the objective of this study was to evaluate the association between the health services offered by primary care teams (PCT) and the detection of new TB cases in Brazil.

## METHODS

This is an ecological study on TB incidence rate, also called as detection rate for the purposes of this study, and its association with the health services offered by PCT in Brazil between 2012 and 2014.

As the incidence of reported cases results from the presence of events in the community (incidence itself) and the sensitivity of the surveillance (detection) system^10^, we worked with the hypothesis that an improvement in the provision of diagnostic services would be associated with greater case detection and, consequently, greater perceived incidence. Thus, for the purposes of this study, we assumed that the rate differences, associated with the actions of the PCT, represent the contributions to the sensitivity of the system more than the changes in the risk of becoming ill.

To measure the provision of health services by PCT, we used data from Module II of PMAQ-AB's second cycle of the “External Evaluation Instrument for Primary Care Teams”^e^. This evaluation was applied to team professionals between November 2013 and June 2014, and data was collected from 29,778 teams in 24,038 basic health units of 5,043 municipalities^d^. In this evaluation, some specific TB questions were only asked to teams that had registered TB cases identified in the previous year to the evaluation.

The study population covered the Brazilian municipalities that registered at least one new TB case diagnosed between 2012 and 2014 and notified in the Information System of Notifiable Diseases (SINAN)^f^, with at least one PCT evaluated by PMAQ-AB's second cycle. Population estimates by municipality were obtained from the Brazilian Institute of Geography and Statistics^g^.

The dependent variable was the TB incidence rate registered from 2012 to 2014, calculated as the ratio of the sum of new TB cases (all forms of the disease) and the estimated population of the municipality of that period. We defined this period based on the timing of the PMAQ-AB evaluation and the availability of data on TB incidence cases at the time of analysis.

The independent variables of PMAQ-AB at the municipal level were analyzed as the proportion (%) of teams that confirmed performing a certain activity in the PC service of that municipality. The variables selected for analysis were those that could conceptually explain the incidence or detection of TB cases in the blocks:

- Institutional support and network support: teams with health surveillance support;
- Territorialisation and reference population: teams with definition of territory coverage; and with territory population not covered by PC;
- Reception and spontaneous demand: teams that conduct risk and vulnerability assessment in patients’ reception;
- Schedule organization: teams with an organized agenda for assistance of spontaneous demands; and with free time in the agenda for follow-up appointments, if necessary, to clarify possible doubts and to evaluate patient's situation;
- Health care: teams with specialized appointment scheduled by the health unit; and that conduct TB active case finding in the general population;
- Exams requested by the PC team: smear sputum for TB; chest X-ray for TB; culture for mycobacteria; serology for HIV; and rapid HIV testing;
- Assistance to the person with tuberculosis: teams that have an annual estimate of TB cases and symptomatic respiratory patients; with first sputum sample for TB diagnosis collected at the first appointment; that have a record of the number of TB identified users in the last year; with the record of TB cases in the service; and that conduct household contact investigation of new TB cases;
- Health Promotion: teams that conduct groups to discuss communicable diseases.

We also analyzed the estimate of the population covered by PC in the municipality in 2013^c^.

Median and interquartile range (IQR) were calculated for each independent variable. These variables were classified as distal and proximal in relation to the outcome. The proximal variables were those that were more directly related to TB detection, such as TB active case finding among the general population and the offer of tests to diagnose the disease. The distal variables were those that represented the organization of the service in general (e.g., record of the number of TB identified users), coverage of the services in the territory (e.g., PC coverage), and offer of services that could be indirectly related to TB detection (e.g., teams that perform groups to discuss communicable diseases).

The analysis was performed with negative binomial regression because of over-dispersion, distribution, and characteristics of the dependent variable. Variables that individually presented associations with p < 0.20 were analyzed in a correlation matrix. In order to avoid collinearity, between variables with correlation higher than 0.40 we selected those with the highest association with the outcome or with the best conceptual support for the multiple model. This process was performed separately for distal and proximal variables.

Following a hierarchical order in the analysis^11^, we obtained a multiple model with the distal variables (model 1). In this model, we kept the variables with p < 0.05. We performed the same process to identify the proximal variables associated with the outcome (model 2). Finally, these latter associations were adjusted by the distal variables of the first model (model 3). This last model was applied in each of the five Brazilian macro-regions to evaluate graphically the variability of the associations.

The measures of association estimated in the regression models were presented as the percentage increasing in the incidence rate ratio (increase in IRR), for each increase in 10 percentage points of the corresponding independent variable. All models included Brazilian States as adjustment variable, because we assumed the existence of variation between the different contexts. However, the regression coefficients per States are not shown in the tables given their extension. We used Stata, version 12 (Stata Corp)^12^, for the analyses.

This study was approved by the National Research Ethics Committee (Process 1.810.520 of November 12, 2016).

## RESULTS

Of the 5,043 municipalities that had at least one PCT evaluated by the PMAQ-AB, 4,428 (79.7% of the total Brazilian municipalities) had at least one new TB case between 2012 and 2014. These municipalities included in the study reported 202,092 new TB cases in the same period (96.4% of all cases), representing an average of 67,364 cases per year.

Regarding the distal variables related to the outcome, the PC coverage was 100% in 63.7% of the municipalities included in this study (percentile 25 of the coverage: 83.1%). Other variables collected in PMAQ-AB also presented a high proportion, such as teams that have a defined territory coverage, that perform the risk and vulnerability assessment in the reception of patients, with network support from health surveillance, that have a record of the number of TB identified users, and that request serology for HIV (Table 1).

**Table 1:** Descriptive analysis and association between distal variables and the detection rate of tuberculosis^a^. Brazil, 2012 to 2014.

Distal variables (every 10%)	Median (IQR)	Increase in IRR (95%CI)
% of coverage of primary care	100 (83.1–100)	-1.88 (-2.87– -0.88)^d^
% of teams with definition of the territory coverage	100 (100–100)	0.21 (-1.75–2.21)
% of teams with population not covered by the primary care in the territory	10 (0–50)	2.23 (1.58–2.88)^d^
% of teams that conduct risk and vulnerability assessment in patients’ reception	100 (87.5–100)	-0.65 (-1.71–0.41)
% of teams with an organized agenda for assistance of spontaneous demands	100 (66.7–100)	1.42 (0.6–2.25)^d^
% of teams with free time in the agenda for follow-up appointments, if necessary, to clarify possible doubts and to evaluate patient's situation	75 (50–100)	0 (-0.7–0.69)
% of teams whose specialized appointment is scheduled by the health unit	40 (0–90.9)	1.64 (1.05–2.23)^d^
% of teams that conduct focus groups on communicable diseases (dengue, tuberculosis, leprosy, HIV, trachoma)	75 (50–100)	0.52 (-0.14–1.19)
% of teams with a network support from health surveillance	100 (80–100)	-0.1 (-0.92–0.74)
% of teams that have a registration of the number of TB cases identified in the last year^b^	100 (100–100)	0.72 (-0.88–2.35)
% of teams that request HIV serology^c^	100 (100–100)	2.8 (1.24–4.4)^d^
% of teams that request rapid HIV testing^c^	87.1 (33.3–100)	0.75 (0.11–1.38)^d^
% of teams that request the rapid HIV testing or serology for HIV^c^	100 (100–100)	2.44 (0.02–4.93)^d^

IQR: interquartile range; IRR: incidence rate ratio

a The measure of association represents the increase in the incidence rate ratio (IRR-1) expressed as a percentage every 10% of the independent variable along with the 95% confidence interval (95%CI). All measures of association are adjusted by States.

b With document that proves it.

c Performed by the network of health services.

d p < 0.05

The following distal variables, adjusted by the Brazilian States, showed a significant association with the TB detection rate: PC coverage, proportion of teams with population not covered by PC, proportion of teams with an organized agenda for reception of spontaneous demand, proportion of teams with specialized appointment scheduled by the health unit, and proportion of teams that request serology for HIV, rapid HIV testing, or any of those tests (Table 1).

The variable “proportion of teams that request serology for HIV or rapid HIV testing” was strongly correlated with “request of serology for HIV” (Spearman 0.65). We decided to include the latter variable in model 1 because it was more strongly associated with TB case detection.

Regarding the proximal variables, the availability of chest X-rays and smear microscopy for TB presented high coverage among the teams. However, activities such as the TB active case finding in the general population (median = 70.6, IQR = 40–100) and collection of the first sputum sample for the TB diagnosis at the first appointment (median = 50, IQR = 22.2–94.4) are not fully incorporated by the teams in the municipalities (Table 2).

**Table 2:** Descriptive analysis and association of the proximal variables with the detection rate of tuberculosis^a^. Brazil, 2012 to 2014.

Proximal variables (every 10%)	Median (IQR)	Increase in IRR (95%CI)
% of teams that conduct TB active case finding	70.6 (40–100)	3.15 (2.46–3.84)^d^
% of teams that request TB X-ray^b^	100 (100–100)	2.43 (1.18–3.69)^d^
% of teams that request smear microscopy for TB^b^	100 (100–100)	3.8 (2.26–5.36)^d^
% of teams that request culture for mycobacteria^b^	100 (71.4–100)	2.36 (1.46–3.27)^d^
% of teams that have the annual estimate of the number of TB cases and symptomatic respiratory cases in their territory	93.8 (66.7–100)	2.19 (1.4–2.99)^d^
% of teams whose first sputum sample for diagnosis of TB is collected at the first visit	50 (22.2–94.4)	0.81 (0.15–1.46)^d^
% of teams that have a registration of the number of TB cases identified in the last year^c^	100 (100–100)	0.8 (-0.62–2.23)
% of teams that conduct household contact investigation of new TB cases	100 (0–100)	3.56 (3.02–4.11)^d^

IQR: interquartile range; IRR: incidence rate ratio; TB: tuberculosis

a The measure of association represents the increase in the incidence rate ratio (IRR-1) expressed as a percentage every 10% of the independent variable along with the 95% confidence interval (95%CI). All measures of association are adjusted by States.

b Performed by the network of health services.

c With document that proves it.

d p < 0.05

Except for the “proportion of teams with registration of TB cases in the service”, the proximal variables were significantly associated with the outcome (Table 2). We did not consider the request of chest X-ray for TB in the multiple analysis, since it presented an important correlation with the request of smear microscopy (Spearman = 0.46), and the latter had a greater association with the registered incidence.

In model 1, the distal variables positively associated with detection were the proportions of teams: with population not covered by the PC in the territory; with specialized appointment scheduled by the health unit; that request serology for HIV; and with an organized agenda for assistance of the spontaneous demand (Table 3). An increase of 10.0% in the proportion of PC coverage was associated with a decrease of 1.9% in IRR (95%CI -3.06– -0.76). In addition, an increase of 10.0% in the proportion of teams that request serology for HIV represented an increase of 2,4% in the studied outcome (95%CI 0.81–3.94).

**Table 3:** Association of the distal and proximal variables with the detection rate of tuberculosis^a^. Brazil, 2012 to 2014.

Variable (every 10%)	Model 1 Adjusted increase in IRR (95%CI)	Model 2 Adjusted increase in IRR (95%CI)	Model 3 Adjusted increase in IRR (95%CI)
% of teams with population not covered by the primary care in the territory	1.87 (1.2–2.54)		1.69 (1.03–2.34)
% of teams whose specialized appointment is scheduled by the health unit and the date is subsequently informed to the user	1.53 (0.95–2.12)		1.33 (0.76–1.91)
% of coverage of primary care	-1.92 (-3.06– -0.76)		-2.24 (-3.35– -1.11)
% of teams that request HIV serology^b^	2.37 (0.81–3.94)		
% of teams with an organized agenda for assistance of the spontaneous demand	1.32 (0.51–2.14)		
% of teams that conduct household contact investigation of new TB cases		3.04 (2.49–3.6)	2.97 (2.41–3.53)
% of teams that conduct TB active case finding		2.07 (1.37–2.77)	2.17 (1.48–2.87)
% of teams that request smear microscopy for TB^b^		1.91 (0.33–3.52)	
% of teams that request culture for mycobacteria^b^		1.61 (0.67–2.55)	1.87 (0.98–2.76)
2Log likelihood	-28400.7	-28280.0	-28207.6

IRR: incidence rate ratio; TB: tuberculosis

2Log likelihood empty model = -31,217.83

a The measure of association represents the increase in the incidence rate ratio (IRR-1) expressed as a percentage every 10% of the independent variable along with the 95% confidence interval (95%CI). All measures of association are adjusted by States and the other variables included in the corresponding model. All associations were statistically significant.

b Performed by the network of health services.

**Figure 1:**
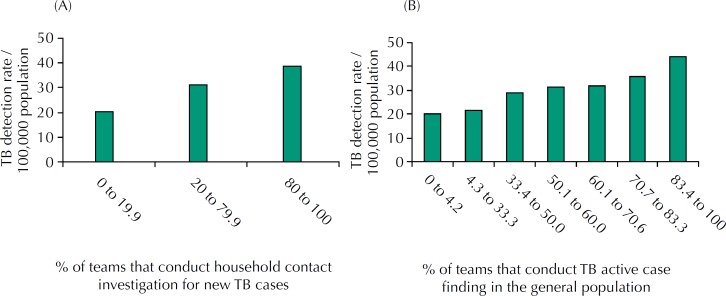
Tuberculosis (TB) detection rate according to the proportion of teams that conduct contact investigation and TB active case finding. Brazil, 2012 to 2014. (A) Household contacts investigation of new TB cases. (B) TB active case finding in the general population.

Regarding the proximal variables (model 2), TB detection rate was positively associated with the proportions of teams that: carry out the household contact investigation; carry out TB active case finding in the general population; request smear microscopy for TB; and request culture for mycobacteria (Table 3). Household contact investigation and active case finding were associated with an increase of 3.0% (95%CI 2.49–3.6) and 2.1% (95%CI 1.37–2.77) in IRR, respectively. The increase in these indicators represented a consistent increase in TB detection rate (Figure 1).

**Figure 2:**
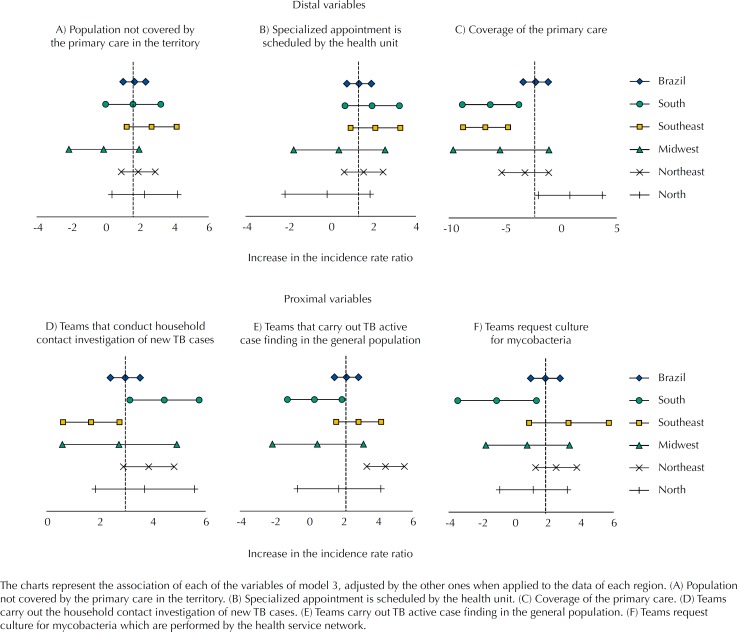
Variables associated with the detection rate of tuberculosis (TB) according to Macroregions. Brazil, 2012 to 2014.

Regarding the final model (model 3), adjusted for the Brazilian States and distal variables, except for the proportion of teams that request smear microscopy for TB, all other proximal variables remained significant (Table 3). Figure 2 presents a description of the associations of model 3, when applied to each of the Brazilian macro-regions.

## DISCUSSION

This ecological study, which included approximately 80% of the Brazilian municipalities, identified the importance of actions performed by PCT and the estimated population covered by PC in the TB case detection. Most of the variables collected by PMAQ-AB are positively associated with case detection, which suggests that PCT activities increase the sensitivity of services. The results highlight the importance of the strategy of the PCT adopted for TB case detection and, consequently, for the identification of the hidden endemic situation in Brazil.

In the distal model, the proportion of teams with population not covered by PC in the territory was positively associated with the registered incidence. In addition, PC coverage was negatively associated with the study outcome. These associations suggest that PC coverage affects the assistance and treatment of persons with TB, reducing the risk of TB transmission. This would influence the outcome analyzed in this study, affecting the risk more than the detection.

On the other hand, variables such as specialized appointment scheduled by the team, the request of serology for HIV, and the organization of the agenda for spontaneous demand were associated with the increase in the detection rate. These actions, although not directly related to TB, indicate that the availability of these actions by the PCT and the organization of these services can favor the diagnosis of the disease and lead to an increase in the sensitivity of the surveillance system.

Regarding the final model, in which proximal variables were adjusted by the distal ones, actions conducted by PCT, directly involved in the TB case detection, were positively associated with the registration of incident cases. This is the case of contact investigation, TB active case finding in the general population, and request of culture for mycobacteria. These results point to the importance of these actions as strategies that should be strengthened to increase case detection.

The variables analyzed in this study were not directly evaluated in previous studies, or at least not in an ecological level^7^ ^,^ ^13^ ^,^ ^14^. However, a study conducted in the South of Brazil has found that health units with FHS had better results for TB case detection when compared to services without this strategy^7^. The FHS has health care characteristics that go beyond the physical barriers of the health units^13^, from the performance of multidisciplinary teams and the coverage of community health agents in the territory^h^. The FHS units are in the PC and, given their relation with the population in the territory, they are in the best position to promote health education intervention activities, identify risk groups, and search for respiratory symptomatic persons^14^.

The adequate offer of supplies and human resources, as well as the request for smear microscopy were positively associated with the detection of cases in PC services when compared to the services of the Emergency Care in Ribeirão Preto, state of São Paulo, Brazil^6^.

A study conducted in Africa has shown that the household contact investigation was more cost effective than the TB active case finding in the general population^15^. However, in Cambodia, Asia, active case finding was more cost effective compared to passive case finding^16^. In this study, these two strategies were factors independently associated with the detection rate, suggesting that they could complement each other to increase the sensitivity of the surveillance system.

The results obtained in model 3 by macro-regions, with some exceptions, showed that most of the confidence intervals of the regional estimates included the estimated for Brazil. Among the exceptions, we can highlight the percentage of PC coverage, in which negative association was most evident in the South and Southeast regions. One of the possible explanations for this variability is that information systems and surveillance sensitivity are better developed in these regions. This could facilitate the measurement of the beneficial effect of the PC coverage on TB incidence.

However, the observed variations between regions could be considered random because they remain compatible with national associations, since no association at regional level reached a statistical significance in the opposite direction to the global estimation. Therefore, the final model would be robust enough to support conclusions at the national level.

Among the limitations of this study, we can mention the impossibility of establishing a chronological order in the independent variables and the outcome^8^. The organization of PC services may be a response to a high incidence of the disease. Otherwise, organized services are expected to be more effective in detecting cases. In addition, the aggregate measures used in ecological studies may differ from the individual ones^17^. From the previous one, we cannot establish a relation between specific PC actions and each of the new TB cases.

Multiple factors could affect the associations found, including socioeconomic variables, which are determinant for the TB incidence^18^ ^,^ ^19^ and the organization of health services^20^ ^–^ ^22^. In this study, we consider that this variability was partially controlled with the adjustment of the Brazilian States in the models. In addition, the models remained relatively stable across regions, thus suggesting that the associations found may be consistent and applicable to different scenarios.

The selection of the teams evaluated by PMAQ-AB is done by adherence and not by probabilistic sampling. The measures of association may have been affected if adherence to the program is associated with the quality of the services offered by the teams. However, of the total number of teams that met the requirements for evaluation in the second cycle^d^, 88.7% were evaluated^i^.

Notwithstanding these limitations, our model was based on national data, from an information system that aggregates all TB notification in the country and the evaluation conducted by PMAQ-AB, the only evaluation program of PC with such a wide scope in Brazil. Our sample of 4,428 municipalities allowed us to evaluate simultaneously the different indicators of PMAQ-AB. In addition, the high variability of our analysis units allows us to extrapolate the results to other locations. On the other hand, the associations of specific strategies and their relation with case detection are consistent with what we expected. Thus, this ecological study provides an overview that can guide the decision making of public policies, especially those related to the strengthening of the public health surveillance system.

In 2015, the WHO estimated that 87% of new TB cases were detected in Brazil^b^, and this estimate was 82% in 2013, the year of this study^j^. The positive association between the services offered by PCT and the identification of TB cases highlights the need to keep an active surveillance system to detect the hidden endemic situation of the disease in the country.

Since 2004, the National Tuberculosis Program (NTP) recommends that the detection and follow-up of persons with TB has to be conducted on PC services^23^. Further efforts should be made to expand PC in the country, as well as incorporate activities to control TB into existing PCT. Lack of adherence to TB control actions can be strongly determined by institutional constraints, as documented in different services^24^. Under the primary care context, these barriers may include high turnover of health professionals, difficulties in attracting qualified physicians to work in remote areas, and the difficulty of accomplishing all territory activities^13^.

This study, which analyzed data from PMAQ-AB and its relation with TB detection, showed that this evaluation tool has a strategic potential to be explored by the different management levels of the Brazilian Unified Health System (SUS). Despite these limitations, future studies should consider it as an important source of data about the situation of services offered by PC teams for TB control and other diseases.

In conclusion, most of the actions evaluated in PMAQ-AB and analyzed in this study are associated with increased sensitivity for TB detection in PC services. However, the estimated population coverage of PC was inversely associated with TB detection rate. This association could represent the overall effect of PC on transmission control, probably from the early detection and treatment of cases.

## Footnotes

a Ministério da Saúde (BR), Secretaria de Vigilância em Saúde. Perspectivas brasileiras para o fim da tuberculose como problema de saúde pública. *Bol Epidemiol*. 2016 [cited 2016 Dec 2];47(13):1-15. Available from: http://portalarquivos.saude.gov.br/images/pdf/2016/marco/24/2016-009-Tuberculose-001.pdf

b World Health Organization. Global tuberculosis report 2016. Geneva: WHO; 2016 [cited 2017 Jan 22]. Available from: http://apps.who.int/medicinedocs/documents/s23098en/s23098en.pdf

c Ministério da Saúde (BR), DATASUS -Departamento de Informática do Sistema Único de Saúde. Informações de saúde (TABNET): assistência à saúde. 2016 [cited 2016 Dec 2]. Available from: http://www2.datasus.gov.br/DATASUS/index.php?area=0202

d Ministério da Saúde (BR), Secretaria de Atenção à Saúde. Programa Nacional de Melhoria do Acesso e da Qualidade da Atenção Básica (PMAQ). Brasília (DF); 2016 [cited 2016 Nov 2]. Available from: http://dab.saude.gov.br/portaldab/ape_pmaq.php

e Ministério da Saúde (BR), Secretaria de Atenção à Saúde, Departamento de Atenção Básica. PMAQ: instrumento de avaliação externa para as equipes de atenção básica. Brasília (DF); 2013 [cited 2016 Nov 15]. Available from: http://189.28.128.100/dab/docs/portaldab/documentos/instrumentos_ae_sfp.pdf

f Ministério da Saúde (BR), DATASUS -Departamento de Informática do Sistema Único de Saúde. Informações de saúde (TABNET): epidemiológicas e morbidade. Brasília (DF); 2016 [cited 2016 Dec 2]. Available from: http://www2.datasus.gov.br/DATASUS/index.php?area=0203

g Ministério da Saúde (BR), DATASUS -Departamento de Informática do Sistema Único de Saúde. Informações de saúde (TABNET): demográficas e socioeconômicas. Brasília (DF); 2016 [cited 2016 Dce 2]. Available from: http://www2.datasus.gov.br/DATASUS/index.php?area=0206

h Ministério da Saúde (BR), Secretaria de Atenção à Saúde, Departamento de Atenção Básica. Política nacional de atenção básica. Brasília (DF); 2017 [cited 2018 Jan 31]. (Série E. Legislação em Saúde). Available from: http://dab.saude.gov.br/portaldab/pnab.php

i Cavalcanti PCS. Atenção básica no Brasil: desafios e iniciativas para qualificação [dissertation]. Brasília (DF): Universidade de Brasília; 2014.

j World Health Organization. Global tuberculosis report 2014. Geneva: WHO; 2014 [cited 2017 Jan 22]. Available from: apps.who.int/iris/bitstream/10665/137094/1/9789241564809_eng.pdf

## References

[1] Esmail H, Barry CE, Young DB, Wilkinson RJ (2014). The ongoing challenge of latent tuberculosis. Philos Trans R Soc Lond B Biol Sci.

[2] Bartholomay P, Oliveira GP, Pinheiro RS, Vasconcelos AMN (2014). Melhoria da qualidade das informações sobre tuberculose a partir do relacionamento entre bases de dados. Cad Saude Publica.

[3] Paiva RCG, Nogueira JA, Sá LD, Nóbrega RG, Trigueiro DRSG, Villa TCS (2014). Acessibilidade ao diagnóstico de tuberculose em município do Nordeste do Brasil: desafio da atenção básica. Rev Eletron Enferm.

[4] Antunes LB, Tomberg JO, Harter J, Lima LM, Beduhn DAV, Gonzales RIC (2016). Sintomático respiratório de tuberculose na atenção primária: avaliação das ações segundo as recomendações nacionais. Rev RENE.

[5] Neves RR, Ferro PS, Nogueira LMV, Rodrigues ILA (2016). Acesso e vínculo ao tratamento de tuberculose na atenção primária em saúde. Rev Pesq Cuid Fundam.

[6] Andrade RLP, Scatolin BE, Wysocki AD, Beraldo AA, Monroe AA, Scatena LM (2013). Diagnóstico da tuberculose: atenção básica ou pronto atendimento?. Rev Saude Publica.

[7] Cardozo-Gonzales RI, Palha PF, Harter J, Alarcon E, Lima LM, Tomberg JO (2015). Avaliação das ações de detecção de casos de tuberculose na atenção primária. Rev Eletron Enferm.

[8] Yirgu R, Lemessa F, Hirpa S, Alemayehu A, Klinkenberg E (2017). Determinants of delayed care seeking for TB suggestive symptoms in Seru district, Oromiya region, Ethiopia: a community based unmatched case-control study. BMC Infect Dis.

[9] Li Y, Ehiri J, Tang S, Li D, Bian Y, Lin H (2013). Factors associated with patient, and diagnostic delays in Chinese TB patients: a systematic review and meta-analysis. BMC Med.

[10] Rothman KJ, Greenland S, Lash TL (2008). Modern epidemiology.

[11] Victora CG, Huttly SR, Fuchs SC, Olinto MT (1997). The role of conceptual frameworks in epidemiological analysis: a hierarchical approach. Int J Epidemiol.

[12] StataCorp (2011). Stata Statistical Software: release 12.

[13] Victora CG, Barreto ML, Leal MC, Monteiro CA, Schmidt MI, Paim JS (2011). Health conditions and health-policy innovations in Brazil: the way forward. The Lancet.

[14] Sulis G, Centis R, Sotgiu G, D'Ambrosio L, Pontali E, Spanevello A (2016). Recent developments in the diagnosis and management of tuberculosis. NPJ Prim Care Respir Med.

[15] Sekandi JN, Dobbin K, Oloya J, Okwera A, Whalen CC, Corso PS (2015). Cost-effectiveness analysis of community active case finding and household contact investigation for tuberculosis case detection in urban Africa. PloS One.

[16] Eang MT, Satha P, Yadav RP, Morishita F, Nishikiori N, Van-Maaren P (2012). Early detection of tuberculosis through community-based active case finding in Cambodia. BMC Public Health.

[17] Schwartz S (1994). The fallacy of the ecological fallacy: the potential misuse of a concept and the consequences. Am J Public Health.

[18] Pelissari DM, Diaz-Quijano FA (2017). Household crowding as a potential mediator of socioeconomic determinants of tuberculosis incidence in Brazil. PLoS One.

[19] San Pedro A, Oliveira RM (2013). Tuberculose e indicadores socioeconômicos: revisão sistemática da literatura. Rev Panam Salud Publica.

[20] Scatena LM, Villa TCS, Ruffino-Netto A, Kritski AL, Figueiredo TMRM, Vendramini SHF (2009). Dificuldades de acesso a serviços de saúde para diagnóstico de tuberculose em municípios do Brasil. Rev Saude Publica.

[21] Brito EWG, Silva AKF, Teixeira GGA, Dias GBS, Costa NDL, Uchôa SAC (2015). Organização do cuidado à tuberculose na atenção básica do Rio Grande do Norte. Rev Enferm UFPE.

[22] Gonzales RIC, Monroe AA, Assis EG, Palha PF, Villa TCS, Ruffino-Netto A (2008). Desempenho de serviços de saúde no tratamento diretamente observado no domicílio para controle da tuberculose. Rev Esc Enferm USP.

[23] Figueiredo TMRM, Villa TCS, Scatena LM, Gonzales RIC, Ruffino-Netto A, Nogueira JA (2009). Desempenho da atenção básica no controle da tuberculose. Rev Saude Publica.

[24] Chapman HJ, Veras-Estévez BA, Pomeranz JL, Pérez-Then EN, Marcelino B, Lauzardo M (2017). Perceived barriers to adherence to tuberculosis infection control measures among health care workers in the Dominican Republic. MEDICC Rev.

